# Making invisible care visible. Ethics and aesthetics of care in participatory arts practices in times of COVID-19

**DOI:** 10.1080/13569783.2022.2147817

**Published:** 2023-01-05

**Authors:** L. de Kock, B. C. Groot, J. Lindenberg, G. Struiksma, T. A. Abma

**Affiliations:** a Leids Universitair Medisch Centrum, Leiden, Netherlands; b Leyden Academy on Vitality and Ageing, Leiden, Netherlands; c Seniorentheater De Rimpel, Leeuwarden, Netherlands

**Keywords:** Ethics of care, aesthetics of care, participatory art, COVID-19, older people

## Abstract

The COVID-19 pandemic emphasises the importance of care for our societies, yet underscores the inferiority of relational caring practices. During this time, we studied the participatory work of artists working with older adults using participant observations, in-depth interviews and visual ethnography. In this article, we present a case study of one arts initiative, a theatre company engaging seniors in the Netherlands, using ethics and aesthetics of care as sensitising concepts. The findings reveal that this work can promote relational forms of care. This study makes visible how different forms of care can be identified in a participatory art project.

**March 2020, the Netherlands**

Silence.

Emptiness.

Dismay.

There was absolutely nothing at first. Like many other countries, The Netherlands went in a lockdown. Care organisations shut their doors for visitors. Older people had to stay at home. Group events were cancelled.

Only about a week before, we had organised a festive kick-off day, where artists, older adults and project managers of 18 different participatory arts projects and participatory researchers gathered to learn from each other’s experiences. Arts in Care was going to be a nationwide participatory mixed-method study to gain insight into the value and mechanisms of participatory arts activities for older adults in the Netherlands. We were interrupted by a pandemic, which forced everyone to change their plans, including us.

After about a week, we picked up the phone and started asking: ‘How are things?’ Sorrow. Uncertainty. Artists were perplexed, taken aback. Many of them did not only have to cancel their activities with older people, but they had lost any opportunity of making an income. Later that year, it turned out the cultural sector was hit hard by the pandemic, with revenues in Europe plunging by approximately 31% compared to 2019, especially impacting smaller businesses and the self-employed (Llehrmitte 2021; Betzler et al. 2020).

Early research into the pandemic's effects on mental health and well-being in the Netherlands (Sociaal en Cultureel Planbureau 2020) shows only a slight drop in people's overall well-being compared to 2019. Yet, this SCP study highlights the rise in specifically emotional loneliness during the pandemic, particularly in the group of people older than 75. These scholars state that, especially during threatening times, people feel a need for close social contact and thus a focus on relational care and wellbeing. During this time, however, safety protocols were given top priority, and focus was given to acute care in hospitals and protocolled medical care in long-term care homes. In short, the understanding of what it meant to care for one another in society, was reduced to a focus on the bare necessities, which meant physical care.

The value of care work, and especially prolonged, chronic care, like the care for older people, is persistently underrated and made invisible, often becoming ‘work to get out of, to pass on to someone who can't get a better job’ (Stuart Fisher 2020, 6). The invisibility of caring practices becomes further obscured when care is performed by others than who we traditionally identify as care professionals, such as artists. Jayne Lloyd (2020, 211) describes artists as having a kind of sensory attunement and aesthetic sensibility, enabling them to facilitate a specific kind of relational care. In line with this, Clive Parkinson (2017, 160) states that the arts go beyond physical care and that ‘it is perhaps artists who are best placed to help us understand what it is to be human, to have a finite life, to seek meaning in frailty and impose order on the chaos of existence’. If we can see artistic practices in this light instead of attempting to evaluate them in medicalised terms, he argues the arts might offer ‘humanistic guidance’ to traditional care workers surrounding ageing individuals (Parkinson 2017, 155). Especially in times of crisis, we thought, we might be able to empirically explore what these type of ideas about the caring practice artists can provide, look like in real life, in times of hardship. Most artists taking part in our research project found ways to engage with the older people they used to work with, despite the pandemic. We decided to follow their progress and analyse their practice to gain insight in how we might regard it as a form of care. Studying the practice in this way gave new insights into the caring mechanisms of this work. Simultaneously, analysing participatory arts practices raised new ways of thinking about care for older people in our societies. During the first months of lockdown in the Netherlands, we asked ourselves: How does this time of crisis and change in care priorities shed light on the way in which participatory artists are able to provide care for older people in our society? How does this practice enhance our understanding of what it means to care for older people? What can we learn about participatory aesthetic practice when we apply an understanding of care ethics to it?

Several scholars write about the unique qualities of applied theatre practices in relation to creative ageing, and how it might develop into a field of its own (McCormick 2017). Research considering these practices is not easy to place in academic boxes such as within the fields of applied theatre, arts and health, arts education, or art therapy. Artistic practice with older people can take place in all of these different fields and also in overlapping spaces in-between, because the goals of different initiatives can vary from medical outcomes to political and social outcomes to personal development and artistic growth. What most of these practices have in common however, is a ‘shared understanding of the benefits of social engagement’ (McCormick 2017, 8). This means that this form of practice often focusses on affect over effect: ‘ … relational applied theatre with older adults considers the importance of the moment of interaction and the affect communally experienced in that moment’ (McCormick 2017, 8). Often, however, great focus is put on the effects, the outcome of these practices, viewed through a medical lens, suggesting that ageing brings with it psychological as well as physical difficulties, which the arts might help overcome (i.e. Fancourt and Finn 2019). However, Parkinson (2017, 148) argues cultural value and the value of participatory arts practices with older people should be understood through its own language. In this article we further this latter perspective and aim to contribute to the body of literature containing empirical and ontological studies considering the unique role participatory arts processes can play in ensuring the social engagement of older people (i.e. Bernard, Rickett, and Pruchno 2017; Bradfield 2021; Groot et al. 2021; McAvinchey 2013; Nicholson 2011; McCormick 2017).

## Ethics and aesthetics of care

We have analysed the empirical findings presented in this article by using two different theoretical concepts: ethics of care and aesthetics of care. In the following two paragraphs we will provide as short exploration of the literature and main definitions of these concepts.

First, it is necessary to gain a basic understanding of what it is to care. Care work and its ethical dimensions can be analysed using an ethics of care framework, as described by Joan Tronto (2013, 1993, 1998). The concept of ‘care’ in this case, refers to ‘everything that we do to maintain, continue and repair our ‘world’ so that we can live in it as well as possible. That world includes our bodies, ourselves, and our environment’ (Tronto 1998, 3). Therefore, care can be seen as an action, an inherently relational practice, never a set standard of principles and rules, and grounded in responsibilities flowing from relationships. It is flexible, depending on the people engaged in the caring practice and their values, way of life, or living conditions. Furthermore, Marian Barnes (2012) describes ‘care’ as a universal human need. Therefore, care can also be regarded as a practice which is important when striving for socially just societies. According to Tronto (1993), a trusting and mutually valued relationship develops in and during a cyclical caring process. She distinguishes four phases and accompanying virtues involved in the process of caring, later adding a fifth, political dimension of care, see Table 1. In this article we will use this framework as a way of freshening our view of the practice of participatory artists working with older people. Correlatively, we look at how this practice raises new ways of thinking about this framework.

**Table 1.** Phases and virtues involved in the process of caring (summarised from Tronto 2013, 35–38).

Phase	Virtue
1. Caring about	Becoming aware of the caring need, which requires attentiveness.
2. Caring for	Assume responsibility for meeting the noticed need for care.
3. Caregiving	The actual task of giving care, which requires knowledge and competence.
4. Care receiving	Taking into account the way in which care is received, being responsive and able to adjust where necessary.
5. Caring with	Viewing caring as political as well as personal, as a necessary practice for social justice. Involves being aware of inequality, conflicts and vulnerability present in the caring relationship, making sure there is trust and respect between caregiver and care receiver.

The second concept we use to analyse our findings is aesthetics of care, which proved to be useful specifically when looking at artistic practice as a form of care. Thompson (2015) introduced the idea of looking at care as a form of aesthetics. He suggests that art and ethics of care are related, starting from the observation that ethics of care focuses on an embodied, relational process, and so do participatory art-making processes. There are two ways of looking at aesthetics of care. Firstly, looking at care work as having an aesthetic quality: ‘ … I propose that in that focused attention between bodies we can recognise an artfulness that is too rarely acknowledged’ (Thompson 2020, 215). Looking at care in this way means that care practices get a relational, sensory quality when whoever is providing care exhibits an artistry in how they perform their caring duties. This is suggested to have a satisfying effect for all those directly involved. A second way of understanding aesthetics of care is to look at participatory art as a practice with caring dimensions. Making art together can then be regarded as a mode of care that exists somewhere between art and social practice (Thompson and Stuart Fisher 2020). In our study we mainly see this latter form of aesthetics of care: we analyse the practice of a participatory artist and examine how we might understand this practice anew by looking at it as having caring qualities.

## Context and methods

This study is part of a more extensive participatory mixed-method study, *Arts in Care* (Groot et al. 2021). The experiences of older adults and other stakeholders in the original research project would be gathered in a participatory fashion, as described by Tineke Abma et al. (2019) and Hilary Bradbury (2015). The research project was meant to be carried out starting January 2020 and finishing one year later. This original study was interrupted by the COVID-19 pandemic. Notwithstanding COVID-19 measures, all 18 different *Arts in Care* initiatives made active arts participation for older people possible in some way during this time. To study these adaptations, experiences were gathered during the first lockdown period of COVID-19 in the Netherlands (March 2020–June 2020) by participant observations and telephone interviews with artists and older participants of art projects.

This article focuses on a critical case example that was deliberately selected as an illustrative case with learning potential (Abma and Stake 2014). We chose the theatre company called *Seniorentheater de Rimpel* as our case study (in English: ‘Senior theatre the Wrinkle’) and the making of their film *Simpel de Rimpel* (‘Simply the Wrinkle’) during the COVID-19 pandemic. We selected this case study because it provided a possibility to examine how an artist adapted her participatory arts work with older people to the COVID-19 mitigation measures, and how she managed to provide forms of care for older people during this time. This project also provided sufficient opportunities for researchers to be present, build up a relationship of trust, observe the work, and have in-depth conversations with participants and the leading artist. This critical case study offered a rich potential to learn more about what care looks like in participatory artistic practice with older people. Taking a phenomenological approach and zooming into first-hand experiences of the researcher, artist and older participants in this case study, we yield an in-depth understanding and illustrate matters which might be overlooked in large, overall studies (Abma and Stake 2014).

## Ethics

This study was reviewed and declared not subject to the law on research involving human subjects by the Amsterdam UMC, location VUmc (IRB00002991) with number 2019.738 at 29-01-2020. The review board decided that the Medical Research Involving Human Subjects Act (WMO) does not apply to this research. The protocol was assessed and considered compliant with scientific due diligence. We asked all participants of the *Arts in Care*-study for informed consent. In this article, we use the real names of artists and participants. For this, they have provided specific consent.

## Seniorentheater de Rimpel

The following section continues in the first-person perspective of the first author of this article, Lieke de Kock to, as explained in the research methods, stay as close as possible to first-hand observations and experiences of the researcher, artist, and participants. Gea Struiksma, the third author and artistic director of *Seniorentheater de Rimpel*. Afke de Graaf, and Frouk de Vries, two of the actors of *Seniorentheater de Rimpel* featured in this article, will be named by their real names, as they have requested. This also contributes to making them and their work visible.

*Seniorentheater de Rimpel* is an amateur theatre company based in Leeuwarden, a city in the north of the Netherlands. The actors of the company are all aged 65 and above, with some of them being 90 + years old. All of them live at home, many of them depending on some form of formal care service or assistance of informal caregivers. The actors are all women. Director and project manager Gea Struiksma leads them in rehearsals every week. Over the course of about five years, she and the actors have built up a working relationship and formed a community.

**Figure 1.**
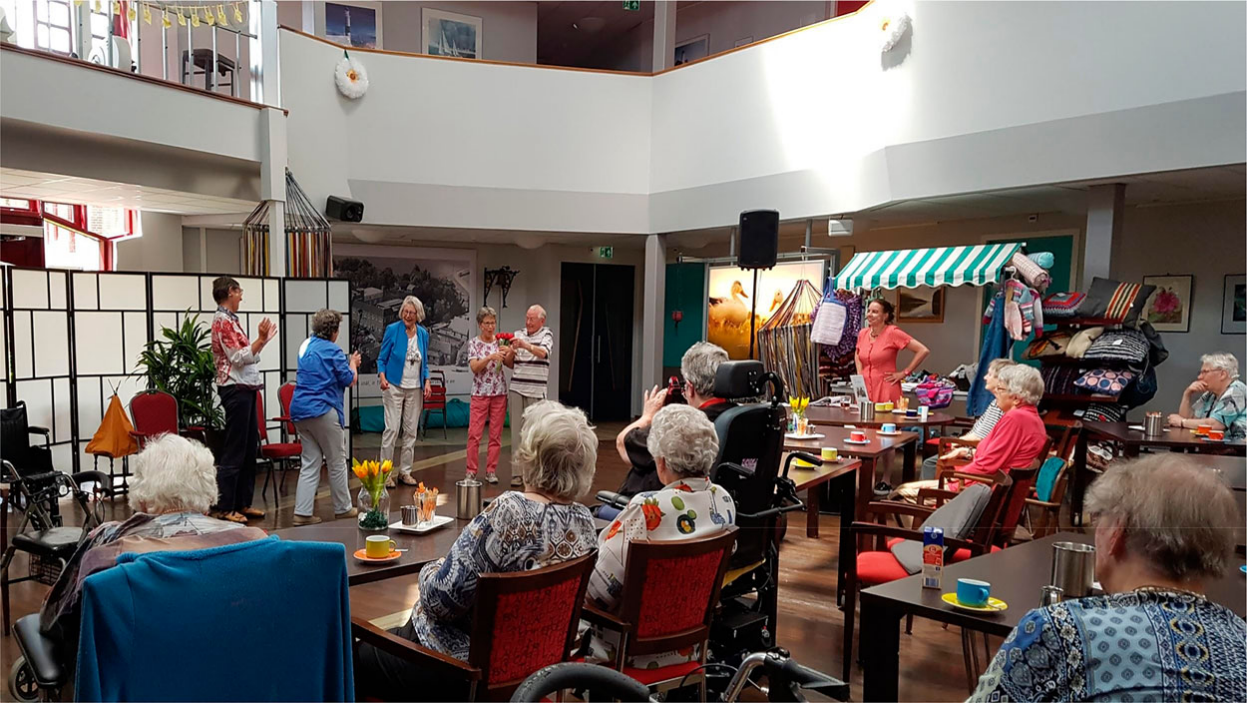
*Seniorentheater de Rimpel* performing in a long term care facility (Photo: Henny Kalisvaart).

The company mainly works through improvisation. The actors work around themes relevant to their lives, which they bring up themselves, such as providing informal care for older family members, loneliness or healthy ageing. They improvise scenes around the topic, which results in a devised performance with more or less agreed upon scenes and storylines, but which remains largely improvised. *De Rimpel* often performs in long term care facilities, during which they invite the audience to take part in certain sections of the performance in what can best be described as a type of forum theatre (Boal 1974): audience members get to give their input or take part in altering the outcome of the play. See Figure 1.

## Staying in touch

The outbreak of COVID-19 put an end to *De Rimpel*’s performances in long term care facilities. Since the actors of *De Rimpel* were themselves part of an at-risk group of adverse outcomes from COVID-19 disease, Gea decided she did not want to risk getting them together for rehearsals, so she cancelled all meetings for the next weeks. Speaking to her barely two weeks into the first lockdown, she explained how she felt despondent and overwhelmed. She described that making theatre with people gave meaning and purpose to her life, together with a sense of connection and community, all of which she was missing now. As an actor and director, she earned a living not just from directing and teaching amateur actors, but also from performing during smaller gatherings and guiding theatrical city tours, all of which had been cancelled. She had hardly any income to look forward to in the next few months.

Despite her personal struggles, Gea explained how she had been in touch with the actors from *De Rimpel* and that social life had stopped completely for a lot of them. Reading groups, exercise classes, everything had been cancelled. Family members had stopped visiting, scared of bringing the virus to their mothers or grandmothers. Gea states: ‘They had very little to look forward to’. She stayed in touch through phone calls, e-mails, and WhatsApp. Most of the older women expressed to her how they missed getting together and having fun in the theatre studio. During a telephone interview, one of the actors told me she kept falling asleep in front of the TV, and she missed rehearsals because they made her feel ‘so awake’. At the same time, Gea, as an artist, wanted to continue making art as for her as well as for the actors of *De Rimpel*, this was a way of giving meaning to life. Rehearsals used to take place every Monday and provided a weekly ‘break’, a time to have fun, as Afke states: ‘On Sundays I’m already thinking, yes, tomorrow is Monday, then we’ll be allowed to play again’. So, Gea decided she needed her actors, and they needed her to create art, especially in times of difficulty.

This process of coming towards the realisation that an artistic intervention was wanted and needed, can be linked to caring about and caring for, when we look at it through Tronto’s ethics of care framework. These aspects, according to Tronto (1998) involve ‘becoming aware of and paying attention to the need for caring’ and taking the responsibility to meet a need once it has been identified, which will become clear when looking at the steps Gea took later. Arguably, Gea paid attention to the caring needs of herself and of the older women in her theatre company in this case. Gea’s becoming aware of hers and the older women’s needs was grounded in a relationship developed in the group over time and through making art together. The way Gea naturally stayed in touch with the women she had known and worked with for several years, can be regarded as ‘caring for’. It can be argued that from a place of relationship, empathy and conversations with the women, a responsibility emerged to engage together in an artistic process.

Gea took responsibility for realising this artistic process by coming up with an alternative way of creating art in times of COVID-19 limitations. She planned to go by each actor’s home individually and work on improvised scenes, little sketches, or parodies about how they were getting through the COVID-lockdown. Then she aimed to record those scenes with the help of a professional filmmaker and hoped they would all be able to gather to watch the big premiere together. Her responsibilities included making plans and gathering funds for a film she was going to make with the older women. She states:

I wasn’t going to sit there talking about how they felt lonely or depressed; I’m not a therapist. What I wanted to do was make art, give them the responsibility of having to come up with creative ideas, get them out of their comfort zone.

Afke and Frouk state that just Gea coming up with this idea already caused a stir in the group of women: they started contacting each other through e-mail and WhatsApp, discussing what was going to happen. Making a film was new to all of these women, and they started fantasising and were curious for each other’s ideas. Here it is possible to identify a sense of solidarity emerging because the theatre project was causing a stir, challenging the women and therefore making them turn to each other for support, the sharing of their concerns, but also their excitement. It provided them with something to talk about, a respite from being isolated and stuck in their apartments because of the lock-down. When relating this to our understanding of caring for or caring about, we see that participatory arts practices can help us see that caring for older people can entail more than protecting them, or making sure they are safe and healthy, which was the general tendency during the COVID-19 pandemic. This practice shows us that providing a challenge, perhaps even a temporary feeling of discomfort or unsafety, can actually enhance a sense of solidarity or community and also provide much needed opportunities for personal growth and development, as we will see when we describe how the project developed further.

I decided to join Gea in this project where possible. In the next sections of this article, I will describe my experiences during two home visits to actors of *Seniorentheater de Rimpel*, Afke and Frouk. I will describe a few events and particular moments in the preparation and filming process, which can be regarded as examples of caring qualities in Gea’s artistic practice. I will then situate these examples in the ethics and aesthetics of care frameworks. Then I will describe the result of the process, the films, and suggest how they might be regarded as caring works of art.

## The domestic becomes aesthetic

Gea visited each of her actors two times in their own homes. During the first visit, she worked on improvised scenes. Devising scenes and making a film with these women can be regarded as the actual caregiving, the third element of the ethics of care framework, which entails the tasks or actions executed which provide care (Tronto 2013). This is where the caregiver needs competence and knowledge: In Gea's case her skills as a director and theatre-maker. The method Gea used to create scenes with her actors was based on using anything and everything in the participants’ immediate environment as a creative impulse for making a scene, a short story. During the visit to Afke's home, there was first a ‘grand tour’ of the small flat, which was filled with personal creations, lifelong collector's items, and photos of friends and family. Everything seemed to have a story, which was told by Afke as Gea listened and encouraged her to elaborate. Gea approached the objects and surroundings of Afke as wholly new and exciting. To me this was interesting, because it seemed to spark in Afke an appreciation of her own direct environment. Gea would point at an object (a miniature drug store), and Afke would reminisce about how she used to go to markets to collect the miniature items and make these herself. Afke later reflected on this process: ‘Gea would point at something and I would think ‘oh that old thing?!’ Then I would start telling her about it, and that made me think, yes, actually everything has a story’.

It also becomes apparent how this form of caregiving has an aesthetic quality to it, as Lloyd (2020, 211) describes how the performative engagement of artists with the processes of caring for objects can establish a model for relational care:

[…] artists can initiate a form of communication that is mediated through an engagement with objects and materials. Artists are able to communicate in this way because they look at the world with attentiveness and care that breaks down what they see.

What Gea’s practice, or participatory arts practice in general in this case teaches us about the care giving element of ethics of care, is that it can entail encouraging the care receiver to look at their regular, direct environments as potential inspiration for a work of art, a story, which gives meaning and importance to those intimate, nearby environments. Especially in times of COVID-19, where more time than ever was spent in those confined domestic spaces, seeing these in this aesthetic way might provide older people with tools to find a sense of fulfilment and meaning in their own homes. When we keep in mind care giving, this thus suddenly illuminates the sense of care that is innate in the otherwise so seemingly common practice of artist’s looking at objects and surroundings as potential for story and wonder.

## Fun and playfulness

I noticed how Gea’s care was received when we were at Afke’s house. First of all, when we arrived at her home, she had been preparing for the visit in the morning: three cups of tea were ready, and she had bought pastries at the local bakery. This to me suggested that this was an event for her, which she also told us: it was a change in activities compared to the long, dull, days she had had during the lockdown so far. Moreover, Afke had been in touch with other participants in the group, and they had been exchanging ideas in preparation for Gea’s home visits. So, here we see that the artistic process was an instigation for a specific type of social contact, which can also be regarded as a form of care: The actors of *De Rimpel* had something to exchange creative, artistic thoughts about, and they all had individual moments of contact and creative interaction with Gea. The importance of social engagement is found in many different forms of creative practice with older adults. In fact, in an overview of developments in the field of research and practice of applied theatre with older adults, McCormick (2017, 8) states that often these projects are ‘built on a shared understanding of the benefits of social engagement’. As opposed to a focus on societal change, education or development, she suggests the value of these practices is often located in the moment of engaging in creative interaction and the affects experienced in that moment. This becomes clear when looking at the interaction that unfolded between Afke and Gea that afternoon. After the first cup of tea, Afke immediately grabbed a knife from her kitchen counter:

I had been thinking, we could make a story about me being hard of hearing. I so often think there is someone in my home but misinterpret the sounds I hear. So I could be experiencing that, and then I would grab this knife and, like, crawl around the house like a police officer, because frankly, sometimes I am inclined to really do so when something scares me!

As she was telling us the story, she was fully committed to the sounds and gestures she would make and showed us these simultaneously. Her eyes lit up, and she spoke with much more intonation than I had heard her do earlier. To me it felt as though she was ‘coming to live’ as it were, as if she was getting caught up in the moment. When Gea encouraged her to think more about the story, she started talking louder and moving faster, getting carried away in the story and her brave, yet cautious protagonist. I saw this as a sign of the woman’s openness and responsiveness to the impulse Gea had given her by asking her to participate in the film. She was receptive to the care Gea provided. According to Tronto (1998), caregiving entails that individuals or organisations perform necessary caring tasks with competence. Care receiving looks at ‘the response of the thing, person or group that received the caregiving’. The caring quality of arts practice, or, linked to ethics of care, the caregiving and care receiving of this project lay in providing a creative impulse, a challenge to set your teeth into, and in the receiver responding to this impulse with joy and excitement. This also becomes clear from the description of the events that followed that afternoon, in the following paragraphs.

**Figure 2.**
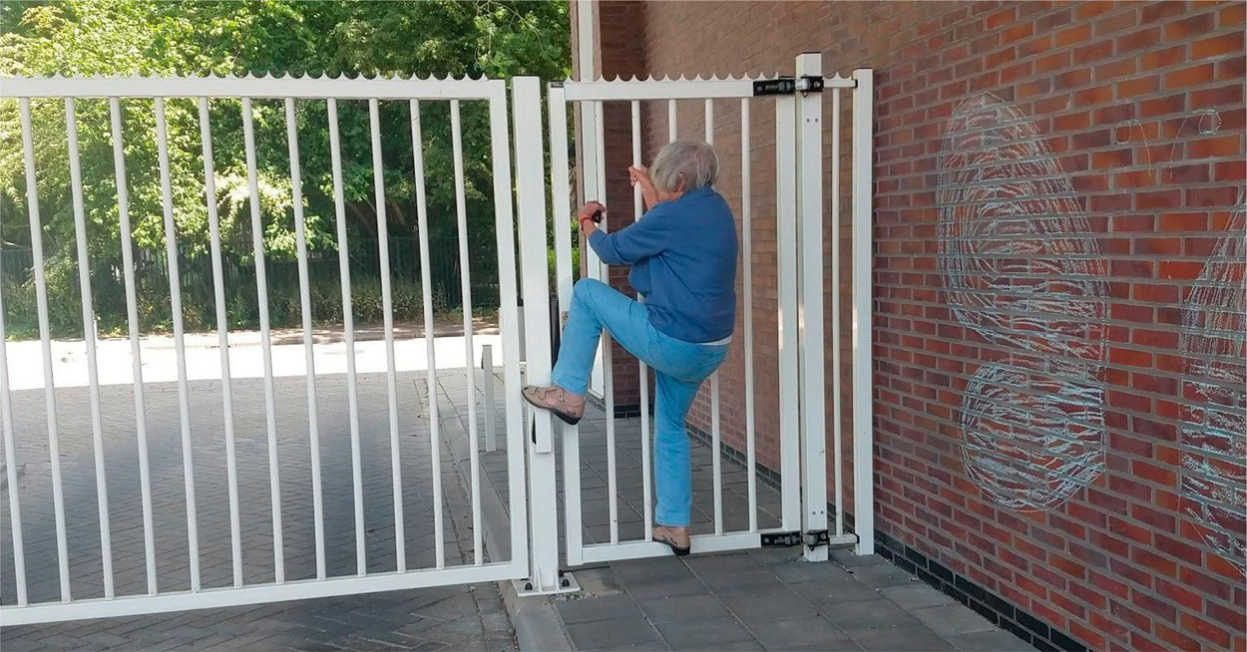
Afke climbing the fence.

**Figure 3.**
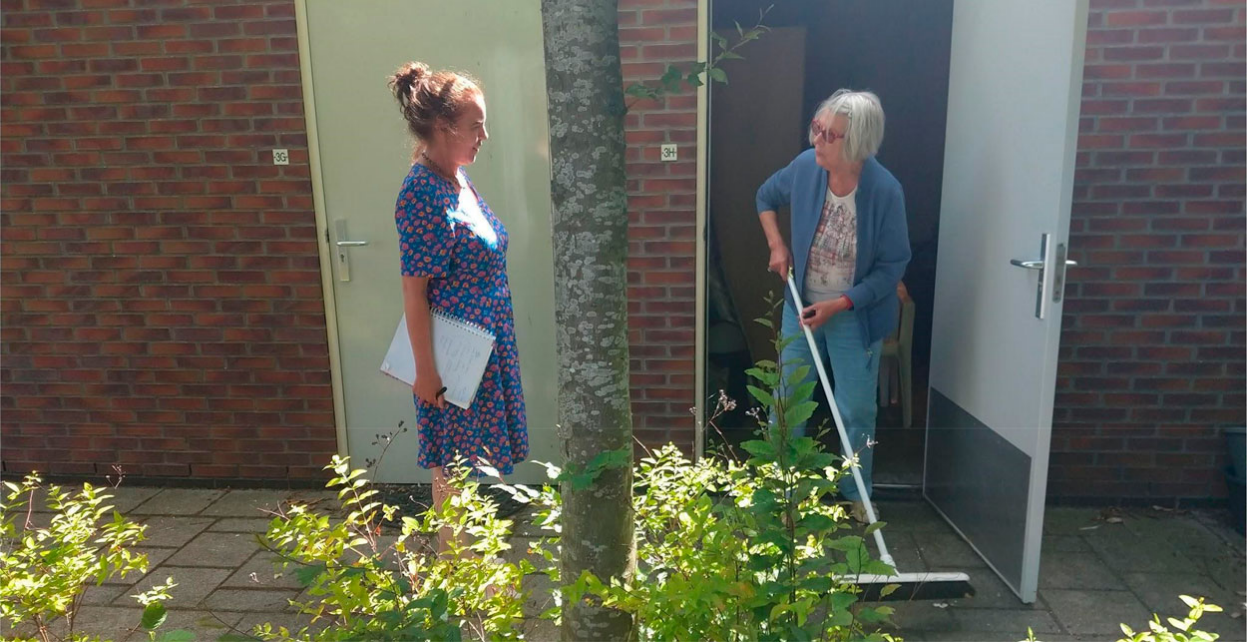
Afke finding a broomstick in her shed.

**Figure 4.**
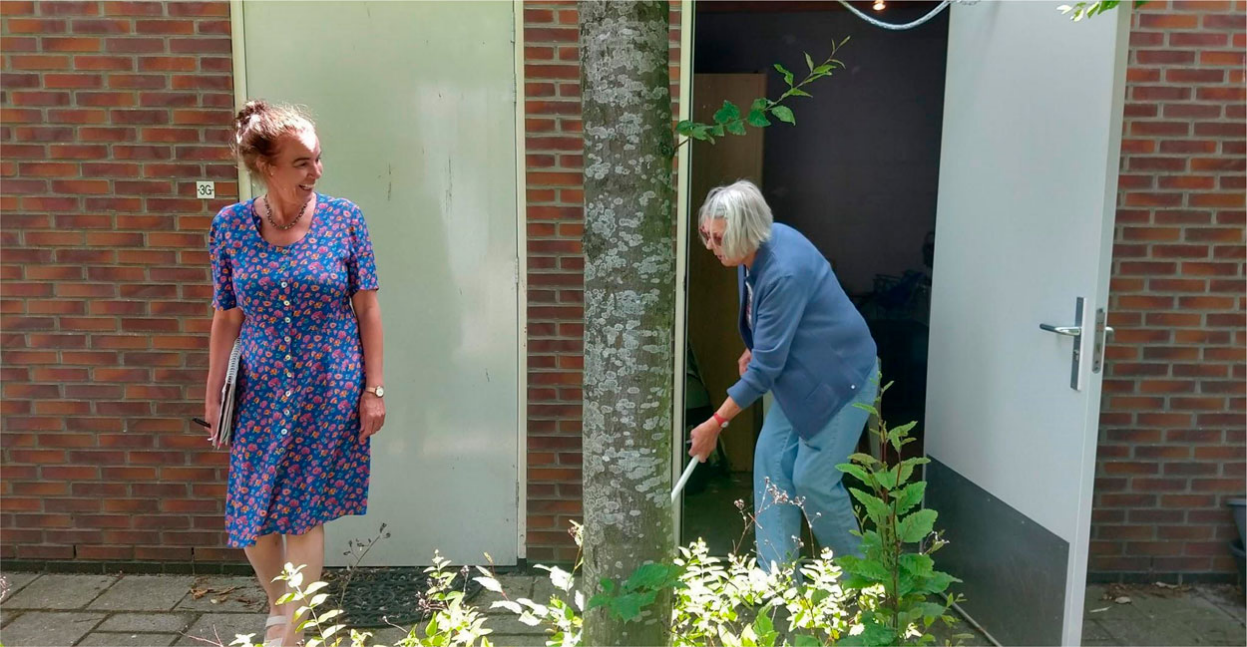
Afke ‘sweeping’ Gea with her broomstick.

What followed was a continuous subtle tuning and aligning in the interaction between Gea and Afke. Gea looked and listened carefully to what Afke offered her in terms of creative ideas, stories or objects from her home, and adjusted her directions and questions accordingly. The process showed how ‘theatre facilitators adopt various performative strategies as a means of inducing and producing a playful and positive emotional state in others’ (Stuart Fisher 2020, 11). Together they got caught up in a kind of game of imagined stories and characters. Gea would ask Afke to go to a particular room or area of her house, or near her house, and encourage her to think of stories that could take place here, characters she could take on and to try acting those out immediately, on the spot. Every time, as the stories came to life, Afke, the actor, came to life, emerged in her role, for instance thinking of naughty things to shout through the fence in her parking lot: ‘I could pretend to be stuck here, as if people had locked me up here, and call out to passers-by, whether they could please help me out. Heck, I could even try climbing the fence!’ (see Figure 2). Alternatively, thinking of all the things she could do with a broomstick found in her shed, like ‘sweeping’ Gea out of her way (See Figures 3 and 4).

**Figure 5.**
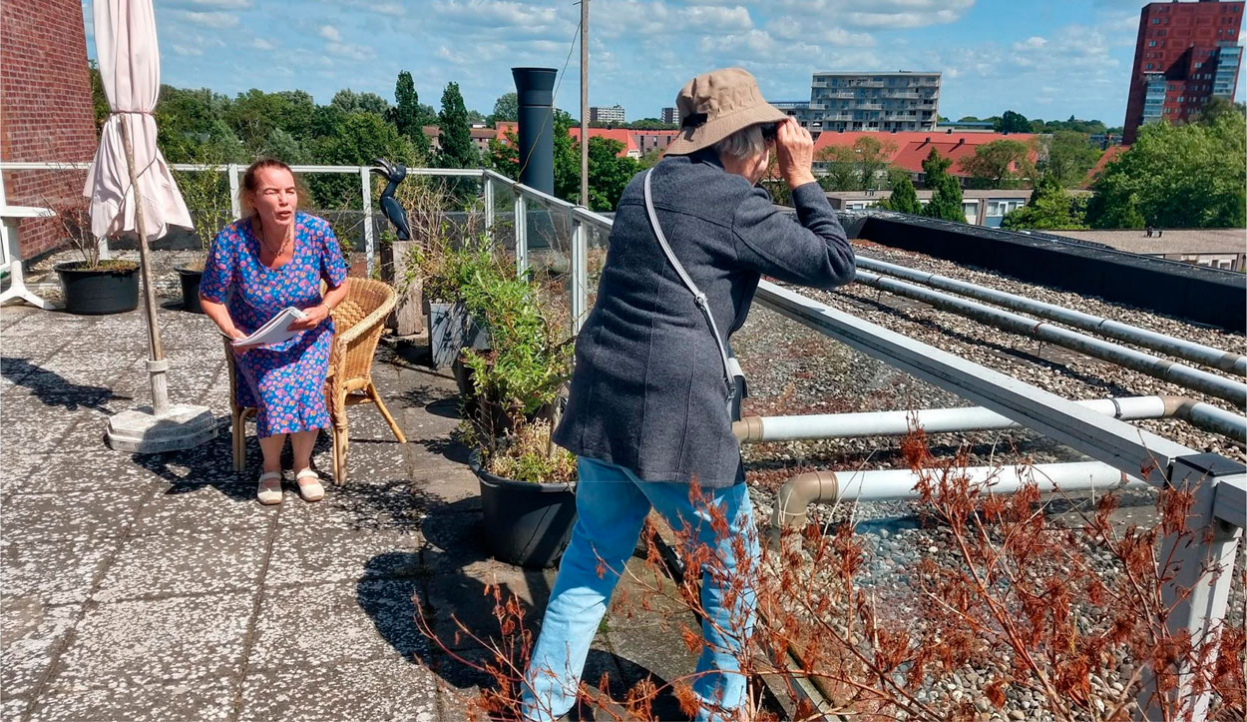
Afke as ‘neighborhood watch’ on her rooftop garden.

Eventually, Gea and Afke ended up on Afke’s communal rooftop garden and settled on a story about her being the ‘neighbourhood watch’, (see Figure 5). Afke explained: ‘Because people have nothing to do these days, they have even more time to watch their neighbours’. It was something she found rather bothersome. She took great pleasure in portraying one of those ‘annoying people’, patrolling the neighbourhood through binoculars (also found somewhere in a box hidden far away in her house), coming up with and writing down silly violations by her neighbours. The part that caused the most laughter and excitement was when she and Gea came up with a hidden side to this neighbourhood watch: they would have their private weed plant hidden on the roof and secretly smoke a joint at the end of the scene when no one was watching. Looking at this creative interaction between Gea and Afke through care ethics, illuminates that care giving and receiving can be a continuous interplay of impulse and response between the carer and the care receiver. The artistic aspect in this case shows that this process can be joyful and playful and create a sense of solidarity in imagining things and acting out stories together.

## Making a film

On another afternoon, I joined Gea and her colleague, a professional filmmaker, on their second home visit to Frouk. The process of coming up with a story had already taken place on a previous visit. From the improvised scene they came up with that afternoon, Gea had created a script and a storyboard. Now, it was time for the actual filming. Frouk told me she was very excited: she had never acted in front of a camera before and was eager to learn how this worked, partly because her grandson was a documentary filmmaker: ‘He will be surprised, I’m sure, to see his grandma be the star of this movie!’ Turning the house into a film studio required quite a bit of preparation: Frouk had a table full of folders, documents, receipts, etc. Just Gea’s request to clear this table, which had probably not been done for years, caused a sort of stir. Slightly worried about not losing things or messing up the organised chaos Frouk had created for herself, she set herself to the task, carefully picking up her belongings and placing them on a different table in the room. Gea later explained how this is what she strives for: to stir things up a little. If we look at this disruptive action Gea encouraged through the ethics of care lens, we can identify that even though this seems like an act of bothering Frouk instead of caring for her, it is also possible to state that this action actually carried in it a form of care. We learn that the caregiving an artist can provide may look like providing a disruption and challenge, however minor in this case. At the same time, it was a form of care which Frouk had not deliberately or consciously asked for, and therefore required trust from her side. Gea states:

Asking something from them which they are not used to, bringing unfamiliar equipment to their homes, bringing a filmmaker and a researcher whom they have never met, gives an impulse of unfamiliarity, a slightly uncomfortable thing to do, which is even in these tiny gestures or requests.

The way in which Gea understood care, as providing disruption and the opportunity to take risks, was radically different from the general tendency in times of COVID-19, when older people were mainly portrayed as vulnerable and in need of protection. When I visited Frouk at a later date, after the film had been finished, I pointed at the table (now filled again) and asked: ‘Remember when Gea asked you to clear that table?’ It made her laugh and reminisce, ‘yes, that table is always full … !’

The story that was being filmed that afternoon was about Frouk’s view from her window. In front of her third-floor apartment, some luscious green trees grew on the square of a former primary school that had recently been shut down. The local council had made plans to cut down these trees and replace them with a parking lot. Frouk commented: ‘They just don’t realize how big an impact this will have on my own and my neighbours’ lives’. Having something green to look at, which changed with the seasons, birds in the trees, was part of what made her want to live in her flat in the first place and made her feel in touch with time and nature. Upon touring her house, she and Gea had found pictures of Frouk demonstrating against nuclear missiles in the eighties. Joined by other women from her family, they would take the bus from Leeuwarden to The Hague (a relatively long travel for Dutch standards, more than 2-hours) to join demonstrations. This is how the film’s story came about: they made up a scene where she prepared to protest against the cutting down of the trees.

In choosing this story to work on, it is possible to recognise several aspects of an ethics of care. Firstly, Gea gets to know Frouk, her personal history and identity, which can be related to caring for. Secondly, Gea and Frouk chose a subject for her scene, which meant something to Frouk in her daily life, about which she wanted to make her voice heard, so the story had the potential for contributing to social justice. Here we can identify what Tronto (2013) describes as caring with. The way in which a drama facilitator works on revealing the stories which the people they work with want to tell is related to politics by Prendergast and Saxton (2012, 9): ‘Helping participants find ways of asking questions that move to the heart of what really interests them is a large part of applied drama – and democracy – in practice’. Enabling Frouk to incorporate her worries, ideas, and experience of participating in demonstrations in the film displays the attentiveness Gea has for her actors’ lived experiences, values, and beliefs. The story shows something of who Frouk is now, where she lives, the objects she surrounds herself with, the ideas she has, and the things she is experiencing. At the same time, it connects to her past and part of her identity and relations, which were formed in joining the nuclear protests with the other women from her family. Looking at this practice through these different aspects of care ethics, highlights that this participatory art project can enhance our understanding of good care. In this context good care entails the care for a person’s life stories, memories and current concerns. Through their joint effort, the artist and the older person create space and time for reflection and appreciation of certain memories or life experiences.

**Figure 6.**
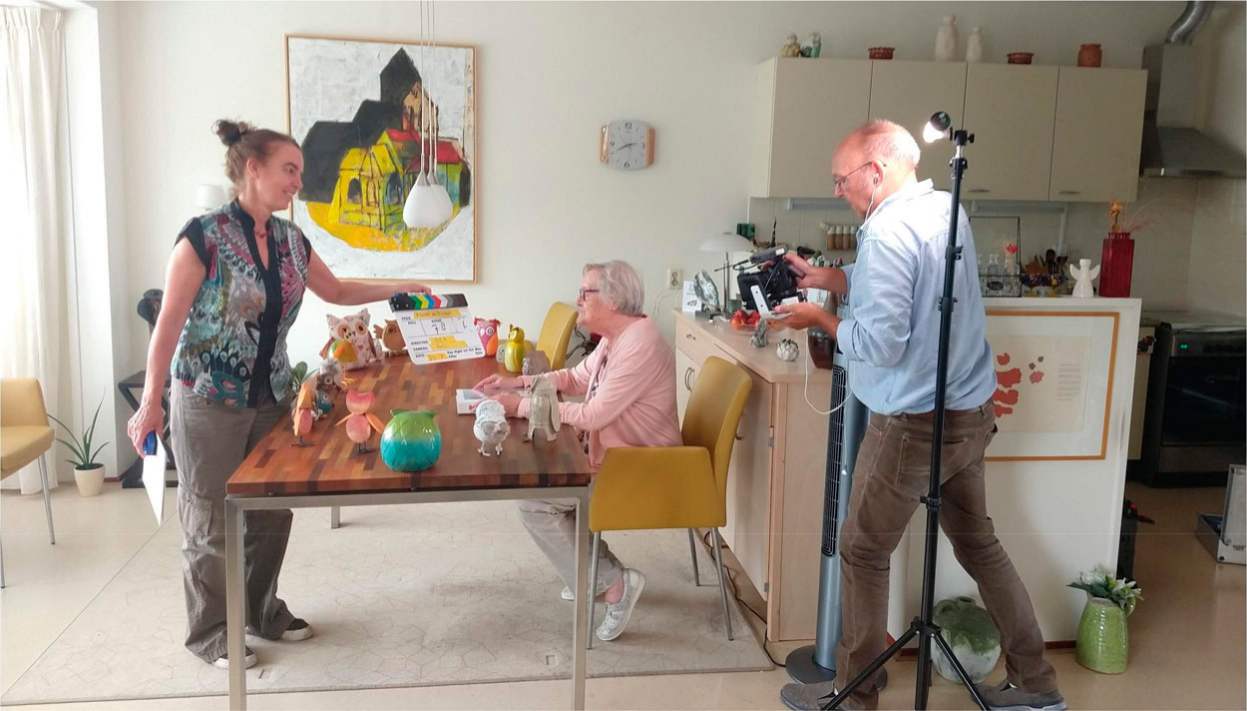
Gea holding the clapperboard, repeating lines before shooting.

During the filming process itself, there was focus, and at the same time, looking through a lens of care ethics, it became clear that the whole process displayed caring qualities. This time, these caring qualities were more soft, not so much challenging or disrupting as Gea had displayed before, but making Frouk feel safe and confident in performing in front of a camera, which was new for her. For instance, before each shot, Gea would conscientiously run through what was expected of Frouk and give her some directions and hints on how to make her character and the story come to life. Frouk could ask any questions she had, or they would go over the scene together as many times as was needed before Frouk would feel confident to start filming. Just before Gea would clap the clapperboard, she would, at Frouk’s request, repeat the lines she was supposed to say during that particular shot (see Figure 6). Many times, Frouk would mix up the lines or improvise and say something completely different. If it came down to the same thing, Gea would leave it at that. If it did not do justice to the story, the shot would be filmed again. The shot would also be repeated when Frouk felt she could do better or wanted another go at this or that particular line or gesture. To me, this process displayed Gea’s care for the aesthetic quality of the work and the way Frouk would look in the final product. Frouk and Afke later jointly reflected and told me:

sometimes you think, I don’t know if this will end up being any good, but well, we do have faith. Because you see, we have always been acting with Gea, and after a while you also know, if it is not good, then she will let you do it again.

When reflecting on this with Gea, she indicated that it was just as important for her that the film would look good, and the older women in it were portrayed with dignity, as it was for Frouk: ‘As an artist, my name is also going to be on the result, on the piece of art. Therefore, it affects me personally if people from my network, for instance, think Frouk looks bad’. This summons questions of co-creation or co-authorship, which are often addressed in discussions on applied theatre ethics (Sadeghi-Yekta and Prendergast 2022). When looking at this art project through an ethics and aesthetics of care lens, it becomes especially important to address these ethical and aesthetical issues. Questions such as: Who is in control of the project? Who makes final decisions? How can power imbalances be minimised and who decides what is ‘problematic’? (Afolabi 2021, 354–355). Throughout the film-making process, the women invited Gea into their homes and thus brought in their own environments and the stories that went with them. However, after improvising the scenes, Gea would go away and write a script for the movie. This raises questions: whose story is it, is Gea tapping into the creativity and aesthetics these women provide her with, and taking the credit for it? She would also ultimately be the one making the final decisions during the creative process. I asked Afke and Frouk how they looked at co-creation within the process and how they saw Gea’s role. They stated that they saw her as their guide and teacher, and they trusted her to make them look good. When I asked whether they found this problematic, or whether they thought there might be a power imbalance, they were adamant, as Afke states:

That is what teachers are for. We are from an older generation. But she also wants us to come up with our own input, and we are always allowed to ask things if we are uncertain. Then she explains to us why she is making a certain choice, and even then, if I don’t completely agree, I trust her to make the right decision, so I do as she says. And that really always turns out good.

Afke states point blank that she sees Gea as her teacher and describes how Gea has taught her for instance how to really tap into anger, and to dare to shout and scold at her fellow actors, without having to be afraid that anyone would take it personally, or to be ashamed of these intense emotions:

She stood opposite me and really shouted and was so angry, I thought, ah, is that what you want?! Well, I can do that too, so I started to really get into it, I was a bit shy afterwards, but Gea said: ‘Yes Afke, this is what I meant, now you’re acting!’ I have felt so much more free after that, and it just feels great every time!

The importance of trust and the building of a working relationship inherent to participatory artistic practice becomes apparent in this statement: the artist and the participants have been working together many years, which means the women have gained a sense of trust in the decisions Gea makes about how to best portray their stories. The fact that Gea states the quality of the final result affects her reputation as an artist personally, does not mean she thinks she will take all the credit for the work. As her, Afke and Frouk all adamantly state they feel the films have been made by all of them, together. Evidently, there is a sense of dependency and power imbalance in the way that Gea and the women work together, but it is not experienced as ‘problematic’ by the older women or Gea.

It is possible to recognise Tronto’s fifth element of ethics of care in the public aspect of this practice: Caring with. This regards care practice as personal as well as political. It requires making sure there is trust and respect between the caregiver and those being cared for, which is apparent in the way Afke, Gea and Frouk talk about each other and the working relationship they have in working towards an artistic product. What participatory arts projects add to our understanding of care being personal as well as political, is the fact that many of these types of projects have a public output, resulting from personal, intimate interactions. An artistic product is put out into the world at the end of it. This is in opposition with the idea that care is mainly about human relations and an intimate, inherently private practice. The film that is being put out into the world here contributes to public debate and our understanding of what it means to care for older people in society, as will become clear from the description of the final results of Frouk’s and Afke’s films in the following paragraphs.

## Caring works of art

Afke’s final result can be viewed here: Burgerwacht, and this is the result of Frouk’s film: De Demonstratie.

I watched these films as soon as Gea sent me them and immediately felt these results, these digital works of art, had something which I could only describe as a caring, respectful quality. They showed ‘[…] how performances can be caring, responsive and attentive’ (Stuart Fisher 2020, 3). The films are not overly dramatic or polished. They are subtle in the way they portray these women’s personal domestic surroundings, with a sense of humour and wit, like the way Frouk talks to her owl collection or Afke pulls out a joint at the end of her scene. Therefore, to me, these films give an intimate look into these older women’s characters, imagination, and lives and show a holistic view of them as people. Respect and trust are related to caring with, making sure you treat people in ways ‘that do not degrade them in their own eyes or the eyes of others’ (Barnes 2012, 24). The characters portrayed in these films have a certain frailty in how they move carefully, or in their sometimes slightly cracked up voices. Simultaneously, the humour and light sense of self-mockery hints at the strength, playfulness, and resilience these women also possess.

The New Dynamics of Ageing project led by the University of Sheffield showed that older women are more often negatively stereotyped than older men, and the production of counter images can counteract the negative impact of these representations (McCormick 2017, 59). Looking at these films might encourage a shift in thinking about older women in a set way, but seeing these women portrayed in this humorous, artful, respectful way, without being explicit or verbal about it, might also provide a more felt, affective, embodied understanding of other ways of looking at old age in our society. In this case, the project might contribute to ways of looking at older women not as fragile and dependent, but as people who have a story to tell, and who can be funny, imaginative, playful and beautiful to look at. As Afke puts it, when I asked her what it was like looking back at the film, a few months after she first saw it: ‘It just makes me laugh’.

## Participatory art and social justice

I reflected together with Frouk and Afke on the way in which Gea’s work might be regarded as providing care for them and their fellow actors. Frouk chose the following words to describe the way in which Gea provides care:

I certainly feel like Gea cares for us in a way, I don’t really know how to say it, but I think the care she provides is actually more important than any physical care. She cares for our spirits, so that we can ‘stay with it’.^1^ We laugh a lot. But then, we make her laugh as well and sometimes we come up with such crazy plans, she never could have made those up herself!

Moral questions of equity are especially pressing in the care for older people, because they tend to be ‘marked’ with the assumption that they need more assistance. Tronto (1998) states:

In fact, many older people do have greater needs to care: healthcare, physical space, transportation, and housing. Yet, as long as these discussions take place in a context in which the elderly are marked as the vulnerable ones in need of ‘special’ care, we miss our opportunity to see caring in old age as part of a flail life process.

Research has shown older people themselves especially value humour, laughter and positive feelings when joining in arts activities (Groot et al. 2021). The unique care giving competencies of participatory artists can therefore also be seen in the fact that they provide older people with the opportunity to have fun, be imaginative and playful, instead of treat them like frail objects which might break with any form of nudging or taking them out of their comfort zones. Which regular care provider ends up pretend-smoking a joint with an older woman on her rooftop garden?

The careful process of acting collaboratively and display of mutual reliance as exhibited in the example of this film project in times of COVID-19 can be regarded as described by Thompson (2020, 218) as ‘[…] the minute building blocks of that more caring, just society’. This relates to Tronto’s (1993) focus on the political aspect of care. Focus on dependency and interdependency is necessary for a socially just society (Barnes 2012). Often, there are power struggles in caring relationships, especially in elderly care, where the care receiver is generally dependent on the caregiver’s skill, knowledge, or capability. Tronto (1998) states it is considered ‘necessary care’. There is also a risk that older people’s caring needs are considered somehow separate from all people’s needs. This causes imbalances in power relations between caregiver and care receiver. In this case study, Gea could be regarded as the caregiver and the older women as the care receivers. However, power relations, when working on making art together, arguably change. Of course, the actors are dependent on Gea’s craft, knowledge of her profession, and the filmmaker's skills. As Afke stated earlier: they see her as their teacher. This automatically suggests a sense of hierarchy where Gea is ‘above’ the actors in terms of power dynamics. The older women would not have gone to make up these stories, come up with these characters, had there not been a reason to do so. Gea provided them with this reason, namely that they had to contribute to a film project. However, at the same time, Gea was dependent on the creative input of the actors, the stories they told her about their life, the actors themselves in order to make a piece of art. Moreover, as she mentioned herself, the project also provided Gea with work and a sense of hope and purpose during times of hardship and uncertainty. It gave her new artistic challenges, a chance to develop her practice and deepen her relations with the actors in her company. As Thompson (2020, 218) states, the aim is

[…] not to deny that dependent relations might be the source of injustice or the unequal exercise of power, but to affirm and seek out human activity where those forms of dependency might become sources for mutual support and solidarity.

This participatory art process, was intersubjective as well as interrelational. Both the artist and the older person needed something from each other and both had their own vulnerabilities. In solidarity and support of each other they produced art. This form of providing care from a sense of mutual support and solidarity is arguably what is so interesting and different when we look at participatory arts practices as a form of good care. It goes against the idea that to care for someone is a one-way action, or can take place according to set principles or rules, but it brings into practice the understanding that humans are relational and interdependent beings (Barnes et al. 2015).

## Discussion

The COVID-19 crisis provides insight into the potential role of participatory arts activities in nurturing caring societies. During this time, a focus on functional, medical aspects of care took over. Priority was given to the protection of older people in society through ordering social distancing and cancelling all group activities. In times when regular care was so drastically reduced to the bare necessities of physical care, the work of participatory artists became especially meaningful to the older people they worked with. The case study described in this article provides an example of how we can use the concept of care ethics in order to make visible and discuss the caring aspects of participatory art practices with older people. These practices can therefore be identified to have added value to traditional approaches to care, because of their aesthetic qualities. Looking at the case study through the lens of care ethics and care aesthetics allows for new perspectives on what arts can bring to our understanding of care for older people.

Looking at this work through an ethics of care lens (Tronto 2013) revealed the different roles and tasks an artist can take in providing good care. We were able to observe how Gea cared *about* older people: she was attentive to specific caring needs such as the need for creative expression, the need to be challenged, learn new things, have a break from daily routines, and stay connected. It became visible that she cared *for* the older women in her theatre company: she felt a sense of responsibility, given their longstanding relationship and the community they had built. She took responsibility for the caring needs she picked up by coming up with an alternative art project which could continue even during the COVID-19 lockdown. In looking at how the artist executed the film project as a form of *caregiving*, we found this to be present in creating improvised scenes and the process of filming. This required the artist’s skills in a continuous process of adapting to the participants’ creative ideas and personal needs. Furthermore, the artist showed responsiveness and sensibility to how the women *received* her care as she continuously adapted her approach to the needs expressed (verbally as well as non-verbally) in the moment. Finally, the artist cared *with*, as power relations in the caring process were interrelated and interdependent: the artist depended on the participants to invite her into their homes, share their stories and be open and willing to play and experiment with creating movie scenes. The participants on the other hand depended on the skills and knowledge of the artist in order to feel supported in telling their stories in a way that was aesthetically pleasing, interesting to watch and would eventually have the potential to contribute to debates around what it means to be an older woman in society, or to change perspectives on what it might feel and look like to provide good care for this generation.

The aesthetics of care lens was used in this article to reveal the potential added value artists and artistic processes can bring to care. Lloyd (2020) describes skills of herself and her art practice as a type of ‘sensory and aesthetic sensibility’, allowing artists to facilitate a particularly beautiful, aesthetic way of relational care for the people they work with. In this case study we saw many ways in which we can identify forms of care in the working relationship between the artist and the older women. The aesthetic aspect of care was in the artist encouraging participants to look at their direct environments with new eyes, as potential for story and beautiful images. The aesthetic qualities of regular old objects they had had their entire lives suddenly came to life, giving them new meaning and importance. This was arguably especially valuable in times of COVID-19, when so much time was spent locked up at home. The artistic process was also an instigation for social contact; the actors were exchanging creative ideas and there were moments of affective, creative interaction with Gea during the improvisation of scenes and the filming process. Care in this artistic sense also meant receiving a creative impulse, a challenge to set your teeth into as well as experiencing joy and excitement in the process. The project also provided a break from routines, an opportunity to be playful and imaginative, instead of treating older women like frail subjects in need of protection. At the same time, the project made this fun, imaginative side of the women visible in the end products. This means the project was caring in more than a personal way, but it also contributes to shifting attitudes in relation to ageing in society (McCormick 2017, 5). Finally, the artistic process showed that participatory arts processes can become places where care is provided from a sense of mutual support and solidarity. Both the artist and the women were dependent on each other and each had their own vulnerabilities in times of crisis: the older women were cut off from family and group activities in order to be protected, the artist’s performances were cancelled and she was cut off from ways of continuing her artistic process. In this project, by working together on the film, they found what Sadeghi-Yekta and Prendergast (2022) describe as ‘space for our shared vulnerabilities’. The way Gea performs her work is in line with what Thompson (2015, 438), describes as projects which exhibit an aesthetic of care:

The execution of a project figured around an aesthetic of care, therefore, relies on building mutual activities of sharing, support, co-working and relational solidarity within a framework of artistry or creative endeavor. Aesthetic value is located in-between people in moments of collaborative creation, conjoined effort and intimate exchange: these are new virtuosities of care that do not rely on the singular display of self-honed skill.

Looking at the empirical findings in this article through an ethics of care as well as an aesthetics of care lens, gave us as a research team the opportunity to reflect on how these concepts speak to each other, what they might reveal about the practice of participatory artists and our understanding of what it can look and feel like to care for older people in our societies. On the one hand, the ethics of care framework revealed the potential roles artists can fulfil in caring processes. This has a descriptive and interpretative function; it allows for a deeper understanding of the ways in which participatory arts practices might be regarded as a form of care. Adding the aesthetic lens to the ethics of care framework, provided a more in-depth hermeneutics of what artists, or a sense of artistry, can add to caring processes, such as a sense of disruption and the opportunity to take risks and the aesthetic and caring attitude towards objects and life stories. Furthermore, participatory arts projects and the artistic products they produce, might contribute to an affective, embodied understanding of what it is like to live as an older person in our society, and therefore provide a unique contribution to public and political debates. Thus, the concepts turned out to be overlapping and sometimes difficult to take apart, but they are certainly complimentary and provide a useful lens and language to make explicit and visible the care that participatory artists can provide for older people in our society and contribute to more socially just and caring civilisations.

Making it explicit that practices like the work of Gea Struiksma can be regarded as a form of care that has added value to how traditional care providers operate, should open up possibilities for giving artists a more prominent, economically supported role in our societies. Looking at Gea’s work through an aesthetics of care lens provided a way of interpreting the value of artistry in caring processes and of giving words to the intimate, affective and beautiful ways in which the older women and the artist worked together. Analysing participatory arts processes in this way, made visible that art is not just something we should offer to older people for ‘fun’ or ‘distraction’, but can be an integral part of the way we care for each other, and in this case, for older people in societies. Right now, a lot of this work in the Netherlands is executed by freelance artists and is dependent on haphazardly funded projects, often leaving the artists underpaid and struggling to make a living. This became even more clear during the pandemic, as many artists were, and still are, out of work. At times of crisis, such as COVID-19, where a focus on standardised, physical care took over, artists can offer a dissenting voice by intimately connecting people through art making processes. Suppose more attention could be paid to the value of participatory art in nurturing caring societies. In that case, the question could be asked as to how we could make supporting artists who carry out this work a more shared responsibility.

## Conclusion

The potential added value artists can have in caring processes became apparent when we looked through an ethics and aesthetics of care lens at the work of a participatory arts practitioner engaging older women in a film project during the first COVID-19 lockdown in The Netherlands. Making explicit and visible the specific care artists can provide for older people, might lead to a more secure position for them within the caring systems that are in place in our societies. Creating beautiful moments of inter-human artful care, as Thompson (2020, 229) states, can then ‘perhaps hint that there is a better mode of being human and living well together’.

## Note

1 Frouk uses a Dutch expression here, which we have freely translated, ‘er bij blijven’, ‘to stay with it’, she means something like, to stay sane, to keep the brain and ourselves fit and to keep participating in social life.
